# The Role of Hyaluronan Treatment in Intestinal Innate Host Defense

**DOI:** 10.3389/fimmu.2020.00569

**Published:** 2020-04-29

**Authors:** Yeojung Kim, Carol A. de la Motte

**Affiliations:** Department of Inflammation and Immunity, Lerner Research Institute, Cleveland Clinic Foundation, Cleveland, OH, United States

**Keywords:** hyaluronan, innate host defense, intestinal epithelium, tight junctions, antimicrobial responses

## Abstract

Hyaluronan (HA) is best known as an abundantly present extracellular matrix component found throughout the body of all vertebrates, including humans. Recent evidence, however, has demonstrated benefits of providing HA exogenously as a therapeutic modality for several medical conditions. Here we discuss the effects of providing HA treatment to increase innate host defense of the intestine, elucidate the size specific effects of HA, and discuss the role of various HA receptors as potential mediators of the HA effects in the intestine. This review especially focuses on HA interaction with the epithelium because it is the primary cellular barrier of the intestine and these cells play a critical balancing role between allowing water and nutrient absorption while excluding microbes and harmful dietary metabolites that are constantly in that organ's environment.

## Hyaluronan

Hyaluronan (HA), also known as hyaluronic acid or sodium hyaluronate, is a polymer made up of repeating disaccharides of N-acetylglucosamine and glucuronic acid (1).

HA is found in many places in our body including most connective tissues, the skin, the vitreous of the eyes, synovial fluid, in the joints, and the umbilical cord (1). HA is synthesized on cell surfaces as a high molecular weight polymer reaching up to 10,000 kDa and is an important component of the extracellular matrix (2, 3). Since HA lacks a protein core, the process of cellular HA synthesis is a unique process as it occurs at the cell membrane, not in Golgi networks where most glycosaminoglycans are usually made (1). Vertebrates have three evolutionarily conserved and highly homologous (55–70% protein identity) HA synthases enzymes (HAS1-3) (4). During the process of HA synthesis, UDP-D-glucuronic acid (GlcA), and UDP-N-acetyl-D-glucosamine (GlcNAC) monomers are added in an alternating assembly to form HA polymers (3). Interestingly, reports suggest that each of these three HAS enzymes may prefer to synthesize different ranges of HA sizes. In general, HAS1 produces a wide range of HA, HAS2 generates large HA (200–2,000 kDa), and HAS3 synthesizes relatively short HA (100–1,000 kDa) (4).

Upon tissue injury or damage, high molecular weight HA is degraded through multiple pathways into small fragments that can act as danger signals (2, 5). The degradation of HA is accomplished by specific proteins including hyaluronidases (HYAL1 and 2) (6), Cell Migration-inducing and Hyaluronan-binding Protein (CEMIP, also known as KIAA1199) (7), transmembrane protein 2 (TMEM2) (8), as well as non-specifically by reactive oxygen species (ROS) (6). Depolymerization can be roughly categorized into three distinct mechanisms. The first one occurs at the local cellular level. HA binds to specific cell surface receptors such as CD44, before being internalized, and degraded within lysosome of cells by hyaluronidases (21). The second pathway occurs at the tissue level. HA present in extracellular matrices it may be degraded to fragments in the extracellular cellular space where the fragments may signal neighboring cells as Damage Associated Molecular Pattern molecules (DAMPs) via multiple receptors, including CD44, TLR4 and TLR2, RHAMM, and Layilin. Free HA fragments may also be generated in the vasculature by platelets using HYAL2 (22). Ultimately HA reaching the vasculature and lymphatics, travels to the liver, kidney, and possibly the spleen for clearance (2). Clearance of HA is mediated by the HA receptor for endocytosis (HARE), and lymphatic vessel endothelial HA receptor (LYVE)-1 (1). The third pathway of HA depolymerization is non-specific, and mediated by free radicals generated under oxidative conditions. This process is promoted by combined action of oxygen and transition metal cations (22). Once HA fragments are generated by catabolism, for example upon tissue injury, they are thought to initiate an innate immune response and induce inflammation (2, 5).

## Current Clinical Uses of HA

HA is currently being used in medical/ patient applications both in HA-containing medical device and as well as treatments. In particular, HA has efficacy as a viscoelastic tool during the ophthalmological surgeries (23), and for viscosupplementation in intra-articular spaces in patients with osteoarthritis (24, 25). Interestingly, for osteoarthritis patients, in addition to the intra-articular injections, the effects from orally delivered HA have also been investigated. Randomized, double-blinded, placebo-controlled clinical trials have proven the effectiveness of orally administrated HA for osteoarthritis in US, EU, and Asia (26, 27). Furthermore, based on HA's ability to promote cellular wound healing, HA based wound-dressings have proven beneficial to patients with burns, trauma, and ulcers (28). Endogenous HA, as well as its degradation products are generated during the process of wound healing and they are capable of inducing fibroblast proliferation and angiogenesis to promote the repair (29).

Although studies of efficacy of exogenous HA treatment effects are currently limited to specific fields, i.e., orthopedics, ophthalmology, and dermatology, results of clinical trials using HA provide evidence that exogenous HA is a safe molecule that may be used in humans (25, 27, 30).

## Innate Host Defense in the Gastrointestinal Tract

The two major physiological functions of the gastrointestinal (GI) tract are digestion of food and absorption of nutrients, electrolytes, and water. The GI tract also provides a major habitat for the body's beneficial commensal bacterial population, or microbiota. The organ's physiologic challenge is to host the beneficial microbial populations while at the same time protecting the host from pathogenic organisms (31). The mucosal barrier of the digestive tract is a key element in mediating this important balance.

The GI tract is an organ system in direct contact with the non-sterile external environment, and one, which continues to protect against the environmental challenges experienced throughout the continuous lumen in the body. To cope with the substantial microbial challenge, the GI tract possesses multiple layers of host defense mechanisms. The first protective layer is a physical barrier of mucus that is produced by, and lies directly on, the luminal side of the epithelium. Mucins form a physical impediment to contact of microbes and are continuously sloughed off to decrease bacterial contact. The mucus lining also retains antimicrobial peptides (AMPs), an additional mode of protection. AMPs are a family of natural antibiotic molecules produced by cells within an organism that can kill bacteria, fungi, viruses, and parasites (32). Defensin proteins are a class of antimicrobial peptides which act against both gram- positive and -negative bacteria. Alpha-defensins are produced by Paneth cells and neutrophils and beta-defensins are expressed by most epithelial cells. The expression of alpha-defensins is constitutive and does not require bacterial signals to be expressed but bacterial stimuli may induce higher levels (33). Several studies have suggested that a defective inner mucus layer may result in closer contact between epithelium and bacteria as well as their products, which could drive pro-inflammatory responses and lead to the development of diseases like ulcerative colitis and self-limiting colitis (34–36).

The second layer is a tightly bound, single layer of cells, called the epithelium. Intestinal epithelial cells arise from the bottom of crypts from stem cells and as they divide and differentiate into specific type of epithelial cells, they migrate toward surface epithelium area (37). Once epithelial cells are differentiated and matured, these cells are gradually turned over through shedding into the lumen and by apoptotic cell death. Importantly, this process occurs naturally without disruption of the epithelial barrier integrity (38). The epithelium controls the flow of water and nutrients from the external to the internal environment of the body. The epithelial barrier is selectively permeable and absorbs nutrients and water, while at the same time, keeping the tight integrity between epithelial cells preventing bacterial invasion. The integrity of the epithelial barrier is regulated by apical junctional complex (AJC). In the intestinal epithelium the AJC is composed of tight junctions and adherens junctions (39). Even though both types of junction complexes are involved in cell-cell adhesions, the functions of each complex are different. Adherens junctions consist of the transmembrane protein E-cadherin, alpha-, gamma-, and delta-catenin and they initiate cell-cell contacts, and maintain the contacts (40). On the other hand, the tight junction is the most apical junctional complex and it is critical in the regulation of permeability in the intestinal epithelium. Tight junctions are composed of transmembrane proteins [i.e., claudins, occludin, and junctional adhesion molecule (JAM)], as well as cytoplasmic scaffolding proteins [i.e., zonula occludens (ZO-1, ZO-2, and ZO-3)] (39, 40). Most tight junction proteins mediate the formation of a tight epithelial barrier. However, there are also reports of tight junction proteins, including Claudin-2, inducing a leaky gut barrier to modulate absorption of ions and water as part of the normal physiology (38). The importance of the functional tight epithelial barrier has been emphasized through the association with diseases. Dysregulation of tight junction proteins have been reported in diseases such as inflammatory bowel disease (IBD) and celiac disease (41, 42). Upregulation of claudin-2 and downregulation of occludin and ZO-1 have been observed in IBD patients (39, 43). Current data suggests that HA can strengthen barrier integrity and therapeutic approaches that enhance epithelial barrier integrity may prove beneficial for patients with gastrointestinal disease.

Beneath the epithelium resides a loose connective tissue, known as the *lamina propria*, largely made up of extracellular matrix, sub-epithelial fibroblasts, smooth muscle cells, and resident immune cells. The *lamina propria* contains a population of leukocytes that provides immune surveillance and protection against invading organisms. Maintaining a healthy, functional mucosa is critical for the prevention of bacterial infections in our gut and many diseases are directly linked to an imbalance in one or more functions of the mucosal barrier (39).

## Exogenous Hyaluronan Treatment and Intestinal Innate Host Defense

In addition to the clinical device uses of exogenous HA treatments for osteoarthritis, wound healing, and in ophthalmological surgery, there are also a number of pre-clinical studies (Table 1) examining the effects of orally administered HA on other organs and diseases (15–17, 44). Overall, oral treatment with exogenous HA has been proven to be beneficial. For example, a recent study has demonstrated that treatment with HA 35 kDa reduces the proinflammatory signaling in Kupffer cells and protects mice from ethanol-induced liver injury by regulating the expression of micro RNA (9, 10). Furthermore, the potential use of intravesical instillations of HA in interstitial cystitis/painful bladder syndrome has also been suggested (11, 29).

**Table 1:** Summary of HA used in pre-clinical models.

**HA sizes**	**Route of delivery**	**Effects**
HA 35 kDa	Oral	Protection from ethanol-induced liver injury *in vitro* and *in vivo* (9, 10)
Undefined	Topical	Treatment for interstitial cystitis/painful bladder syndrome (11)
HA 750 kDa	IP	Proliferation of colonic epithelium *in vivo* (12)
HA 750 kDa	IP	Protection from DSS-induced colitis *in vitro* and *in vivo* (13)
HA 750 kDa	IP	Protection from irradiation *in vivo* (14)
HA 35 kDa	Oral	Induction of an antimicrobial peptide *in vitro* and *in vivo* (15)
HA 35 kDa	Oral	Decreases bacterial infection *in vitro* and *in vivo* (16–18)
HA 35 kDa	Oral	Increases the expression of a tight junction protein *in vitro* and *in vivo* (16, 17, 19)
HA 35 kDa	Oral	Reduce intestinal permeability in DSS-induced colitis mouse model (16)
HA 35 kDa	Oral	Protection from NEC model *in vivo* (20)

*HA molecular mass and route of administration appear to be important for specific efficacy*.

Multiple studies have shown a variety of effects of HA treatment on intestinal epithelium. Riehl et al. has shown that long-term (5 weeks) intraperitoneal administration of HA 750 kDa induces the proliferation of colonic epithelium in healthy mice (12). Additional studies have revealed that the HA receptors, CD44 and TLR-4, mediate the proliferative phenotype of colonic epithelium post HA treatment *in vivo*, although *in vitro* the same report has shown that HA 750 kDa treatment does not alter proliferation of intestinal epithelial organoids (45). On the other hand, Zheng et al. have demonstrated that the same treatment protects mice from dextran sulfate sodium (DSS)-induced colitis when the HA treatment was started at the same time as DSS treatment (13). In that study, exogenous HA treatment induces the expression of tumor necrosis factor α (TNFα), macrophage inflammatory protein-2 (MIP-2), and cyclooxygenase-2 (COX-2) in a MyD88-dependent manner in mouse peritoneal macrophages *in vitro* and in the distal colon *in vivo* (13). Even though Zheng et al. have shown that COX-2 is induced in macrophages as a result of HA 750 kDa treatment, another study has shown that HA 200 kDa treatment has no effect of COX-2 expression in HIEC cell line (human normal small intestine cell line) (13, 46). Additional studies by Riehl et al. have also shown that intraperitoneal HA 750 kDa treatment 8 h before irradiation is radioprotective and increases crypt survival and diminishes radiation-induced apoptosis in proximal jejunum of mice in a TLR-4 dependent manner (14). Taken together, all these studies suggest that while intra-peritoneal delivery of HA in mice modulates intestinal epithelium indirectly, it directly affects macrophages in the *lamina propria*. Plausibly, changes observed in epithelium may be dependent on the HA-affected immune cells present in *lamina propria*.

In addition to exploring the role of intraperitoneally administered HA, the effects of oral delivery have also been investigated. Hill et al. have shown that oral administration of specifically HA 35 kDa, but not significantly larger or smaller sizes, induces the expression of beta defensin-2, an antimicrobial peptide, in intestinal epithelium *in vitro* and *in vivo* through TLR-4 (15). Furthermore, Hill et al. has reported that HA isolated from human milk also increases the expression of beta defensin-2 *in vitro* and *in vivo via* CD44 and TLR-4 as well as inhibits *Salmonella enterica* infection *in vitro* (18).

Oral HA35 has shown protective effects in *in vivo* bacterial infection models as well. HA 35 kDa treatment has been reported to decrease severity of murine *Citrobacter rodentium* infection, a model organism which is similar to enteropathogenic *E. coli* in humans (16). Both recoverable *C. rodentium* CFU (colony forming units) and epithelial bacterial translocation were reduced in these studies. In this same report, HA 35 kDa treatment increased the expression of a tight junction protein zonula occludens-1 (ZO-1), a critical component in forming tight junction complexes between intestinal epithelial cells that prevents bacterial infection (16). In agreement, HA 35 kDa mediated ZO-1 induction has been shown to act directly on mouse epithelium *in vitro* (19). Accordingly, oral gavage with HA 35 kDa also diminishes the observed increase in intestinal permeability post DSS treatment of mice (16). Recently, Kessler et al. have shown that oral treatment with HA 35 kDa inhibits *Salmonella* infection *in vivo* and decreases the expression of Claudin-2, a leaky tight junction protein (17).

In two additional models of colitis which are also bacterially driven, the dextran sulfate sodium (DSS) (13) and the murine necrotizing enterocolitis (NEC) model (20), oral HA35 was shown to be protective. HA 35 kDa treatment diminishes the observed increase in intestinal permeability post DSS treatment of mice (16). Interestingly, oral gavage with large sizes of HA such as 2,000 kDa does not induce expression of either beta defensin-2 or ZO-1 in mouse intestinal epithelium. Clearly, intraperitoneal injection of large HA 750 kDa and oral treatment of small HA 35 kDa have obvious beneficial effects on intestinal epithelium while oral treatment of large HA 2,000 kDa has no effect. Moreover, the result of a study using HA isolated from human milk (milk-HA), which contains 95% of HA ~500 kDa and 5% of HA ~35 kDa, indicates that orally administered 500 kDa size of HA or combination of both 500 kDa and 35 kDa HA may be effective for protecting intestinal epithelium (18, 47). Hill et al. has shown that milk HA is a more potent inducer of defensin-2 than HA 35 kDa, since a lower concentration of milk HA (up to 700-fold less) is sufficient to obtain the effects comparable to HA 35 kDa *in vitro* and *in vivo* (18). Although requiring further studies, it is provocative to speculate that HA 2,000 kDa may be simply too large to physically cross the mucus layer present on the intestinal barrier.

In a model of NEC, mouse pups receiving HA35 had increased survival and lower intestinal injury compared to untreated NEC. HA 35 also reduced intestinal permeability, bacterial translocation, and proinflammatory cytokine release in NEC pups as well as upregulating epithelial tight junction proteins claudin-2,-3,-4, occludin, and ZO-1, suggesting that HA 35 protects against NEC at least partly by enhancing epithelial barrier defenses (20).

## HA Sizing Effects on Intestinal Epithelium

A still unanswered question that remains is “why are HA effects frequently size specific?” In theory, each HA receptor recognizes a 6–10 sugar residues of HA and this should be the case independent of the HA length (48). However, multiple studies have described the effects of HA to be size specific (3, 9, 15, 49).

Based on the studies demonstrating that HA effects may be obtained only from a small-, but not a large size HA, one of the possible explanations may be the inability of certain size of HA to reach the cell surface and to bind the HA receptors expressed there. Moreover, before HA reaches the cell surface, environmental components including extracellular matrix and mucus might affect the capacity of HA to bind to cell surface receptors. For example, while HA is given orally, to be able to bind to HA receptors on the intestinal epithelial surface, HA must pass through the mucus layer. The mucus is comprised of many highly glycosylated proteins and it is possible that a large HA such as HA 2,000 kDa may be unable to reach the epithelial cells. Kessler et al. show that oral HA 35 kDa indeed makes its way to the colon and comes in contact with epithelium (17).

**Figure 1:**
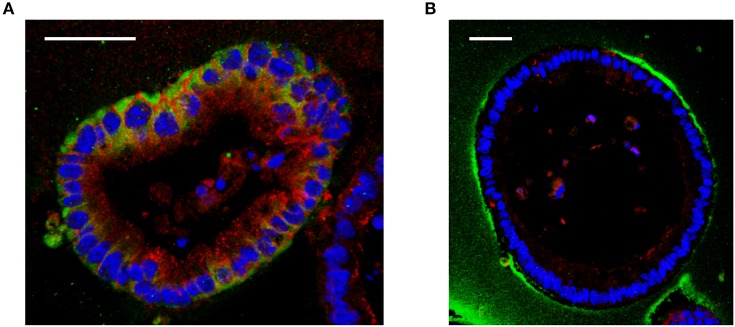
Effect of different sizes of HA on cultured mouse intestinal organoids. **(A)** Immunofluorescent staining of HA 35 kDa (350 micrograms/ml, 30 h) treated mouse intestinal organoids show internalized HA (*green*) and increased ZO-1 expression (*red*) compared to **(B)** HA2000 kDa (350 micrograms/ml, 30 h) treated mouse intestinal organoids, which show no uptake of HA (*green*). DAPI = blue. Scale bar = 50 mm. Immunohistochemical staining for HA was performed using biotinylated HA binding protein. Z-stack images were obtained using confocal microscopy and 3D images were generated using Velocity.

Interestingly, although the large HA is able to reach the surface of the cells *in vitro*, it is not internalized by cells unlike a small size HA 35 kDa (Figure 1) (19). These data indicate that the internalization of HA is size dependent and may not occur with large sizes of HA. Absence of CD44 receptors in mouse intestinal epithelial cells may be another possible explanation (19). This, however, requires further investigation, as in mouse lung epithelial cells, which express CD44, similar size specific effects as those found by Kim et al. were shown by Forteza et al. (49) Multiple studies have investigated binding of HA to CD44 receptors and have shown that larger HA binds to CD44 with higher affinity than the smaller size HA (50, 51). Future studies are needed to address the question of whether CD44 or additional receptors may be involved in the internalization of HA in a size dependent manner and whether the internalization of HA is required for induction of any downstream signaling pathways.

## HA Receptors in Intestinal Epithelium

High molecular weight HA and fragments of HA can bind to several HA binding receptors including the CD44 receptor, the receptor for HA-mediated motility (RHAMM), HA receptor for endocytosis (HARE), lymphatic vessel endothelial HA receptor (LYVE1), layilin, and toll-like receptors (TLRs-2 and 4) (2) Some investigators have proposed that the signal transduction activated by HA is dependent on the cell-type dependent available HA receptors, cooperatively and/or clustering of HA receptors (3). As mentioned, CD44 is the most highly investigated HA receptor and biochemical studies have shown that larger sizes of HA bind to CD44 with higher affinity (50). Interestingly, the clustering of CD44 upon binding to high molecular weight HA binding can be inhibited by small HA oligosaccharides (51).

Several studies have indicated that toll-like receptors (TLRs) are capable of signaling in response to HA and many of the HA effects have been suggested to be through TLR-2 and TLR-4 (5, 52, 53). Interestingly, for monocytes to respond to HA, TLR-4, and CD44 must form a complex together with MD2 (lymphocyte antigen 96) (54). However, to date, HA has not been demonstrated to directly bind to TLRs.

Among all HA receptors identified, the least studied HA receptor is layilin. Layilin is a transmembrane membrane protein, first identified as a HA receptor in 2001 by *Hynes* group (55). Layilin contains C-type lectin domain, which is similar to the link domain, also known as a HA binding domain (56, 57). Layilin plays important roles in cell migration and membrane ruffling as demonstrated by layilin knockdown inhibiting cell migration *in vitro, and inhibiting* cancer cell metastases *in vivo* (58). Furthermore, layilin is essential for induction of epithelial-mesenchymal transformation (EMT) induced by TNF (tumor necrosis factor)-α in kidneys (59). In addition to HA, cytoskeletal proteins, including talin, merlin, and radixin also bind to layilin, however, the function of this binding has not been investigated (55, 60). Only a few studies have been conducted investigating the effects of HA through layilin. Forteza et al. has demonstrated that in lung epithelial cells expressing layilin, HA 35 kDa or smaller HA, treatment decreases E-cadherin expression while larger HA does not (49). In the report from Kim et al. HA 35 kDa but not HA 2,000 kDa was shown to increases the expression of ZO-1 through layilin while HA 35 kDa treatment does not change the expression of E-cadherin in colonic epithelial organoids (19). The differing results of E-cadherin expression post HA treatment between lung epithelial cells and intestinal epithelial organoids may be due to the fact that layilin is expressed in colonic epithelial organoids in the absence of CD44, while both receptors are expressed in lung epithelial cells (19, 49). Additional studies investigating the significance of the layilin in receptor mediated HA effects on various cells are warranted, and may provide further insight into its cellular signaling and functions.

Although multiple types of HA binding receptors have been identified the specific HA receptors expressed in human or murine intestinal epithelium and the location of HA receptors present have not been clarified. The majority of studies investigating HA effects have been conducted using HA receptor knockout mouse models, which do not directly prove that any of the observed effects of HA requires binding of HA to any of its receptors (15, 19, 45) Studies have shown that in normal human colon CD44 is expressed in the crypts, while mature intestinal epithelial cells do not express CD44 (61). Interestingly, in the mouse, CD44 shows the same expression pattern as human from proximal to transverse colon but distally, CD44 expression is lost even in the crypts (19). Most studies that have been done using human colon tissues have not specified the region within the colon from where samples taken, so we do not as yet know whether there is a regional difference in CD44 expression in human colon, similar to what we observe in mouse colonic epithelium. While TLR-4 is expressed on the cell surface in immune cells, it is also expressed intracellularly in a mouse small intestinal epithelial cell line (62). In human and mouse colonic epithelium, TLR-4 is intracellularly expressed in fetal colon during gestation, however the expression is significantly reduced postpartum (63). Low expression of TLR-4 in colonic epithelium in human colon has been reported in another study comparing colon tissues of normal and IBD patients (64). On the other hand, the regional differences in expression of TLR-4 have not yet been addressed either in human or mouse colonic epithelium. Certainly interesting, and potentially important, is understanding where HA receptors are expressed in the intestinal epithelium. So far, only CD44 has been investigated in a limited number of studies. Limited data indicates that CD44 is expressed in basolateral side of intestinal epithelium in human and mouse tissue which suggests that the effect of ingested HA would not be directly mediated by CD44 (45, 65). Still, we cannot exclude the possibility that the location of CD44 can be changed to an apical location in the presence of HA. Figure 1 suggests that HA receptors bind to exogenous HA might be located apically. Unfortunately, the location of layilin could not been shown due to the technical limitation of detection antibodies. Further studies using the microinjection of HA in spheroid-cultured intestinal organoids could help to address these questions.

## Conclusion

**Figure 2:**
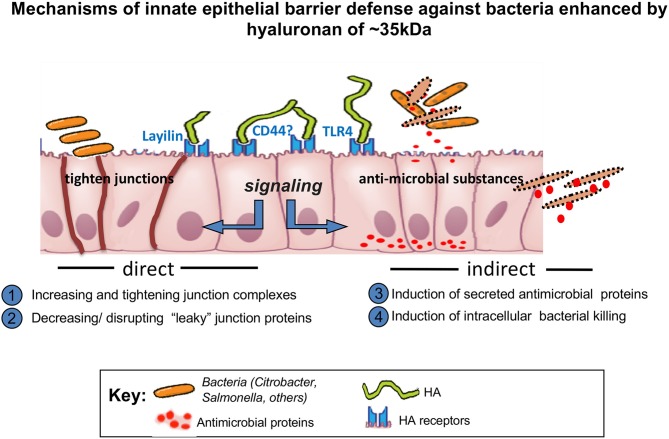
Schematic depicting the activity of HA 35 kDa.

HA is a natural product and is present in human milk at the highest levels immediately post-partum, which supports the notion of its participation in process of maturing the neonatal digestive system. A number of pre-clinical studies highlight functions of exogenous HA promoting intestinal epithelial defense mechanisms and inhibiting bacterial infections in the intestine. Effects of exogenous HA treatment on the intestine have been investigated in multiple studies looking at oral and intraperitoneal delivery *in vivo*. In the case of orally delivered HA, HA 35 kDa appears to be the most potent size of HA for protection of intestinal epithelium (Schematic-Figure 2), different from large size of HA. In the case of intraperitoneally delivered HA, size dependent studies have not been reported, with only HA 750 kDa being used for the intraperitoneal injection *in vivo* (12). In this context, the detailed mechanisms of how certain specific range of HA sizes may be effective in colonic epithelium especially in the *in vivo* setting have not been identified. The complex environment in the intestine including the presence of mucus layer and microflora may be one of the main reasons of why it is difficult to recapitulate the *in vivo* setting using the *in vitro* models. Further studies dissecting mechanisms of how HA treatment promotes defense in the intestine will be helpful to advance any possible future clinical trials utilizing HA treatment to improve intestinal health.

Through the numerous clinical trials of HA intra-articular injections and oral delivery for osteoarthritis patients, as well as clinical usages of HA in ophthalmology, the safety of using HA as a therapeutic modality appears to be most promising. A recent pilot study has also shown that HA 35 kDa is safe for human oral consumption (66). This is not surprising, since many successful and minimally toxic compounds have been of natural origin; the use of probiotics for treatment of colitis is a good example of beneficial use of natural products (67). Recent studies have revealed an underappreciated feature of HA, as a promoter of the epithelial defense mechanisms against pathogens in the intestine (16, 17). We envision HA has the potential to be a novel supplement and prophylactic treatment for pre-mature infants who cannot be breastfed, as well as patients who may have a dysregulated intestinal barrier and who are at increased risk for enteric bacterial translocation.

## Author Contributions

The review was outlined and jointly written by YK and CM.

## Conflict of Interest

The authors declare that the research was conducted in the absence of any commercial or financial relationships that could be construed as a potential conflict of interest.
